# Radiotherapy is enhanced by CPH:SA IL-1α microparticles in a murine HNSCC tumor model

**DOI:** 10.1186/s12885-025-13995-3

**Published:** 2025-04-01

**Authors:** M. M. Hasibuzzaman, Rui He, Ishrat Nourin Khan, Aliasger K. Salem, Andrean L. Simons

**Affiliations:** 1 Interdisciplinary Graduate Program in Human Toxicology, University of Iowa, Iowa City, IA USA; 2 Department of Radiation Oncology, University of Iowa Hospital and Clinics, University of Iowa, Iowa City, IA USA; 3 Holden Comprehensive Cancer Center, University of Iowa Hospital and Clinics, University of Iowa, Iowa City, IA USA; 4 Department of Pharmaceutical Sciences and Experimental Therapeutics, College of Pharmacy, University of Iowa, Iowa City, IA USA

**Keywords:** IL-1, Microparticles, HNSCC, Radiotherapy, Immunotherapy, T cells

## Abstract

**Background:**

Radiotherapy (RT) can trigger immunogenic cell death which may be exploited to improve the effectiveness of immunotherapy. However, recent results from clinical trials testing RT/immunotherapy combinations in head and neck squamous cell carcinoma patients (HNSCC) have been disappointing. Interleukin-1 alpha (IL-1α) is a cytokine that can activate various aspects of anti-tumor immunity including dendritic cell (DC) activation which is critical for the recruitment of tumor infiltrating lymphocytes. Here we test the cytokine IL-1α encapsulated in 20:80 1,6‐bis‐(p‐carboxyphenoxy)‐hexane:sebacic acid (CPH:SA) copolymer-based microparticles (IL-1αMPs) as an adjuvant to RT in a murine syngeneic HNSCC mouse model. Thus the main research objective of this current study was to evaluate if IL-1αMPs can enhance the antitumor immune response of radiotherapy.

**Methods:**

Activation of immune cells in response to RT ± human recombinant IL-1α was evaluated in human peripheral blood mononuclear cell (PBMC):cancer cell co-cultures. A bilateral HNSCC tumor syngeneic mouse model was used to monitor mEERL tumor growth and immune cell recruitment in response to RT (8 Gy to irradiated tumor only) with and without intraperitoneal delivery of IL-1αMPs.

**Results:**

Results showed that IL-1α induced the activation of monocytes, NK cells, T cells, and DCs in PBMC:Cal-27 cell co-cultures but there was no enhanced immune cell activation (with the exception of NK cells) in vitro when combined with RT. RT and RT + IL-1αMPs significantly suppressed growth in irradiated mEERL tumors compared to control. However, only the combination therapy was able to slowdown growth of the non-irradiated tumors compared to the other treatment groups. Immune cell profiling revealed that RT caused acute lymphodepletion on treatment day 3 which was reversed by treatment day 11 in RT-exposed mice. The anti-tumor effect of RT + IL-1α was accompanied by significantly increased infiltration of DCs in the irradiated tumor and increased CD8 + and antigen (E7)-specific CD8 + T cell infiltration in both irradiated and non-irradiated tumors. The anti-tumor response of the combination therapy was completely abrogated by CD8 + T cell depletion.

**Conclusions:**

This data suggests that the addition of CPH:SA IL-1αMPs to RT may boost anti-tumor immune response and target both local and systemic disease. This combination is worthy of further investigation as an immunotherapeutic strategy and could represent a promising approach to improve survival outcomes in HNSCC patients.

## Background

Head and neck squamous cell carcinomas (HNSCCs) are cancers that develop in the squamous cells lining the tissues of the head and neck region. These include the mouth, throat, nasal cavity, sinuses, lips, and salivary glands. According to the latest GLOBOCAN estimates (2020), HNSCC is the seventh most common cancer worldwide [1]. Each year, approximately 890,000 new cases of HNSCC are diagnosed, accounting for about 4.5% of all cancer diagnoses globally. The disease causes around 450,000 deaths annually, which is about 4.6% of global cancer deaths. The majority (~ 60%) of the patients with HNSCC were found at locally advanced stage, which accounts for 400,000 deaths annually worldwide [2, 3]. Radiation therapy (RT) in combination with systemic therapy is the primary non-surgical treatment option for these patients. Although local control is achieved through intensive treatment strategies, risk of developing secondary tumors is significantly high [4]. Approximately 50% of these patients develops local recurrence and there are no curative treatment options for recurrent and/or metastatic (R/M) HNSCC patients. Hence, the effort to understand RT associated oncogenic signaling pathways and to develop therapy targeting those pathways is ongoing. Particularly, the epidermal growth factor receptor (EGFR) targeted therapy has gained significant interest after observing benefits in overall survival (OS), progression-free survival (PFS), and locoregional control (LRC) when patients were treated with combination therapy of cetuximab, and RT compared to RT alone [5]. However, in a phase III randomized clinical trials cetuximab-based chemoradiotherapy was found to be significantly worse in terms of OS, PFS, and LRC compared to cisplatin based chemoradiotherapy in unselected HPV-positive oropharyngeal carcinoma patients [6]. Moreover, addition of cetuximab with cisplatin based chemoradiotherapy treatment regimen did not improve the outcomes compared to cisplatin and RT [7]. These data demonstrate the necessity to develop novel therapeutic strategies to improve patient outcome beyond what is observed with RT with standard chemotherapy or targeted systemic therapy.

RT is an effective local therapy for solid tumors due to RT-induced DNA damage which leads to tumor cell death through senescence, apoptosis, and necrosis. Additionally, RT triggers immunogenic cell death (ICD) which releases new antigens, damage-associated molecular patterns (DAMPS) and cytokines leading to the recruitment and maturation of antigen-presenting cells (including dendritic cells (DCs)), and subsequent priming, activation and influx of cytotoxic T lymphocytes (CTLs) resulting in a potential anti-tumor immune response [8]. The role of immune response as a mechanism of action of RT has led to a plethora of clinical trials studying the combined effects of RT and checkpoint inhibitors (i.e. anti-PD1, anti-CTLA4) [9–11]. Despite RT in combination with checkpoint inhibitors being a promising strategy in theory, it appears that this strategy does not improve survival outcomes beyond current standard of care RT protocols for locally advanced (LA) HNSCC [12–16]. One reason for these failures is due to RT-induced ablation of in-field tumor-infiltrating lymphocytes (TILs) and TILs in the tumor-draining lymph nodes (TDLNs) [17]. A second reason for these failures is that absent or limited pre-existing DC activity may impede on the ability of RT and checkpoint inhibitors to trigger an anti-tumor immune response [18].

Interleukin-1 alpha (IL-1α) is a pro-inflammatory cytokine, which have been studied in several preclinical and clinical studied due to its ability to activate immune effector cells and trigger anti-tumor immune responses [19–21]. However, dose-limiting toxicities including cytokine storm and hypotension has limited its use in the clinic as a cancer therapy [22]. Our previous work showed that the pro-inflammatory cytokine interleukin-1 alpha (IL-1α) could increase DC maturation and activation and showed promise as an immunotherapeutic agent for HNSCC therapy [19]. IL-1α triggers the activation of the IL-1 pathway which plays a critical role in the regulation of immune and inflammatory responses to infections and sterile insults [23]. The IL-1 pathway is triggered when the ligands IL-1α and IL-1 beta (IL-1β) bind to the IL-1 receptor type 1 (IL-1R1) leading to the recruitment of the co-adaptor protein MyD88, IL-1 receptor-associated kinases (IRAKs) and TRAF6, which are important for prompting the expression of target genes involved in immune response [23]. Due to undesired dose-related side effects (i.e. hypotension) associated with systemic delivery of recombinant IL-1α [24], we encapsulated IL-1α in a CPH:SA (1,6‐bis‐(p‐carboxyphenoxy)‐hexane: sebacic acid (20:80)) polymer and previously reported that these CPH:SA IL-1αMPs (IL-1αMPs) released IL-1α in a slow and sustained manner, with no toxicity or loss in bioactivity in HNSCC tumor-bearing mice [19]. The IL-1αMPs also triggered the expansion and activation of CD4 + T cells, cytotoxic CD8 + T cells and natural killer (NK) cells in addition to DCs, suggesting the induction of a broad anti-tumor immune response [19]. Here we will examine if IL-1αMPs will enhance tumor response to RT and further examine changes in immune response in a murine syngeneic HNSCC mouse model.

## Methods

### Cell lines and reagents

Cal-27 and FaDu HNSCC cells were obtained from the American Type Culture Collection (Manassas, VA, USA). SQ20B HNSCC cells were obtained as a gift from Dr. Anjali Gupta (Department of Radiation Oncology, University of Iowa, IA, USA). The HNSCC lines were mycoplasma and human papilloma virus-negative and cultured in Dulbecco's Modified Eagle's Medium (DMEM) with 10% fetal bovine serum (FBS) and 0.1% gentamicin. The mEERL (murine oropharyngeal epithelial cells stably transformed with HPV E6 and E7 together with hRas and luciferase) cell line was obtained from Dr. Paola Vermeer (University of South Dakota, South Dakota, USA) and were cultured in DMEM/Hams F12 with 10% FBS, 0.1% gentamicin, 0.005% hydrocortisone, 0.05% transferrin, 0.05% insulin, 0.0014% triiodothyronine and 0.005% epidermal growth factor. All cell lines are adherent, were cultured in vented flasks at 37 °C and 5% CO_2_ in a humidified incubator and were not used beyond 10–12 passages. Recombinant human and murine IL‐1α (rIL‐1α) were purchased from BioLegend (San Diego, California) and both used at a concentration of 50 ng/mL for 24 h. Anti-hIL-1α, and anti-hIL-1β were purchased from Invivogen (San Diego, California) and used at 1 μg/mL. Anakinra (ANA/IL-1RA) was purchased from the inpatient pharmacy at the University of Iowa Hospitals and Clinics and was used at 10 μg/mL.

### ELISA and clonogenic assays

FaDu, Cal-27 and SQ20B cells were exposed to X-ray radiation using an Xstrahl CIX3 cabinet irradiator at 0, 2, 4, or 8 Gy with a dose rate of 1.1 Gy/min. Cell culture media was harvested at 24, 48 and 72 h following radiation for analysis of levels of IL-1α, IL-1β, IL-6 or IL-1RA using Human Duo Set ELISA kits (R&D Systems, Minneapolis, MN) according to the manufacturer’s protocols and. A Synergy H1 Hybrid Multi-Mode Reader (BioTek, Winooski, VT) was used for colorimetric analysis. For clonogenic assays, cells were irradiated in 60 mm culture dishes. Following radiation cells were immediately trypsinized, counted and plated in fresh media at a concentration of 100–400 cells and incubated for 7–10 days. Colonies were then fixed with 70% ethanol and stained with Coomassie blue and counted.

### In vitro co-cultures and immune cell activation

Human peripheral blood mononuclear cells (PBMCs) were collected from healthy donor blood (DeGowin Blood Center, University of Iowa Hospitals and Clinics) by density gradient centrifugation using Ficoll paque. For the co-culture experiments, Cal-27 cells were exposed to 0, 2, 4, or 8 Gy, grown overnight then PBMCs were added at a 3:1 PBMC:Cal-27 ratio. The co-cultures were treated with human rIL-1α at 50 ng/mL for 24 h with PBS as a control. PBMCs were then harvested and stained with a cocktail of fluorochrome conjugated antibodies (Biolegend): CD45‐PE‐Cy5, CD3‐PE‐Cy7, CD19‐Pacific Blue, CD4‐Alexa Fluor 594, CD8‐PerCP, CD56‐APC, CD14‐PerCP‐Cyanine5.5, CD11c‐BV421, HLA‐DR‐APC‐Cy7, BDCA‐4‐PE, CD123‐Alexa Fluor 700, CD40‐BV605 and CD69‐FITC. Cells were then analyzed on a 5-laser Cytek Aurora Cytometer using FlowJo software (BD Biosciences). The gating strategies were as follows:

- NK cells: CD45 + CD3 − CD19‐CD56 + 
- Activated NK cells: CD69 + CD45 + CD3 − CD19‐CD56 +
- T cells: CD45 + CD3 + CD19‐CD4 + /CD8 + 
- Activated T cells: CD69 + CD45 + CD3 + CD19‐CD4 + /CD8 + 
- Monocytes: CD45 + CD3 − CD19 − , CD56 − HLA‐DR + CD14 + 
- Activated monocytes: CD40 + CD45 + CD3 − CD19 − , CD56 − HLA‐DR + CD14 +
- pDCs: CD45 + CD3 − CD19 − , CD56 − HLA‐DR + CD11c- CD123 +
- Activated pDCs: CD40 + CD45 + CD3 − CD19 − , CD56 − HLA‐DR + CD11c- CD123 + 

The percentage of positively stained cells was calculated and plotted as fold change compared to control.

### Fabrication and characterization of MPs loaded with rIL-1α

Interleukin-1α-microparticles (IL-1αMPs) were produced using a double emulsion solvent evaporation method, as described previously [22, 23]. Briefly, 100 μL of 1% PVA solution containing 500 μg of murine rIL-1α was made. CPH:SA 20:80 polymer (200 mg) was dissolved in dichloromethane (1.5 mL). The rIL-1α solution was added to the polymer under sonication at 60% amplitude for 30 s using a Qsonica sonicator with an ultrasonic converter probe (CL-18, Fisher Scientific) to obtain the primary emulsion. The primary emulsion was immediately transferred to 8 mL of 1% PVA solution and sonicated for 60 s under the same conditions to get the final emulsion. The final emulsion was mixed with 22 mL of 1% PVA and stirred for 2 h to evaporate the organic solvent. MPs were centrifuged, washed with nanopure water, resuspended in 10% sucrose solution, frozen at − 80 °C, then freeze-dried. Empty MPs were prepared using the same process without the input of rIL-1α. Size distribution and zeta potential of the MPs resuspended in nanopure water were determined using a Zetasizer Nano-ZS (Malvern) through dynamic light scattering. To assess the IL-1α loading in MPs, MPs were degraded with 0.5 N NaOH, neutralized by HCl to pH 7.0, then centrifuged at 5000 × g for 5 min. Empty MPs were synthesized the same as the IL-1αMPs, but without IL-1α loading. The amount of IL-1α was quantified using a BCA assay.

### In vivo mouse model

C57BL/6J male mice (The Jackson Laboratory), aged 4–6 weeks were housed in the Animal Care Facility at the University of Iowa (UI), handled using aseptic procedures, and allowed to acclimate for at least 5 days before handling. Food and water were readily accessible to the mice. Approval for all animal procedures was obtained from the Institutional Animal Care and Use Committee (IACUC) at UI and all animal procedures complied with the guidelines set by the National Institutes of Health. The mEERL HNSCC cells (1 × 10^6^ cells/100 μL PBS) were inoculated subcutaneously into both the right and left flank of each animal as a bilateral tumor model. When tumors became palpable (~ 4–5 mm in any direction), tumors on the right side (irradiated) only were treated with a single dose of 8 Gy x-ray radiation at 3.22Gy/min using an Xstrahl Small Animal Radiation Research Platform (SARRP) at the University of Iowa—Ionizing Radiation Services core facility on Treatment Day 1. During the radiation time mice were anesthetized using (100/10 mg/kg) ketamine/xylazine and shielded with 3-mm lead coffins. Twenty-four hours after RT, mice were administered IL‐1αMPs (equivalent to 7.5 μg rIL‐1α in 18.75 mg MPs) or equivalent amounts of Empty MPs intraperitoneally (i.p.) on Treatment Day 2 and Day 10. SHAM mice were administered saline (100 μL) as controls. For the immune cell depletion experiments, murine anti-CD8 mAb (clone 53–6.7) was purchased from BioXcell. Bilateral mEERL-bearing male C57BL/6 mice (*n* = 10–12 mice/group) were administered RT + IL‐1αMPs with or without anti-CD8 (300 µg/mouse). For CD8 + T cell depletion anti-CD8 mAb was given 10 days after the tumor inoculation (when tumors become palpable) twice per week and throughout the course of study. CD8 + T cell depletion in vivo was validated using flow cytometry. Weight and tumor measurements (using Vernier calipers) were evaluated periodically. Tumor volumes were calculated using the formula: tumor volume = (length × width2)/2. The maximal tumor size permitted by the IACUC approved protocol was 15 mm (or combined 30 mm for bilateral tumors) in any dimension. The maximal tumor size/burden was not exceeded in these studies; and mice were euthanized via CO_2_ gas asphyxiation when tumor diameter reached 15 mm in any dimension.

### Tumor and lymph node immune cell infiltration

On Treatment days 3, 11 and 19, subsets of animals (*n* = 3–4) were euthanized to collect tumors (irradiated and non-irradiated) and draining lymph nodes (DLNs, inguinal lymph nodes on the side of irradiated tumor). Tumors and lymph nodes were prepared in single‐cell suspensions and after live/dead staining, incubated with murine antibody cocktails such as: CD45‐Alexa Fluor700, CD3e‐BUV737, CD4‐PerCP, CD8α‐APC‐Cy7, CD11b‐PE‐Cy5, CD11c‐KIRAVIA Blue520, CD19‐BV785, NK-1.1-Pacific Blue, F4/80‐PE‐Cy7, Ly‐6G‐PerCP‐Cy5.5, Ly‐6C‐BV711, and MHC class II‐BUV563. Antigen (HPV)-specific T cell responses were detected by staining with the HPV E7‐specific iTAg tetramer PE‐H‐2Db HPV 16 E7 (RAHYNIVTF). A murine FcR blocker was utilized to prevent nonspecific antibody binding. T cells were defined as CD45 + CD19 − CD3 + CD4/CD8 + lymphocytes; NK cells were defined as CD45 + CD3 − CD19 − NK-1.1 + , Monocytes were defined as CD45 + CD3 − CD19 − CD11b + Ly6C + , Dendritic cells were defined as CD45 + CD3 − CD19 − CD11C + MHCII + , and Macrophages were defined as CD45 + CD3 − CD19 − F480 + immune cells. Cells were analyzed by flow cytometry. Immune cell staining was analyzed and quantified as described above for the in vitro studies. The number of tumor-infiltrating immune cells was normalized to 100 mg of tumor tissue.

### Statistical analyses

For the in vitro studies, one‐way analysis of variance (ANOVA) with Tukey post‐tests was used to detect differences between 3 or more treatment groups. For the in vivo studies, treatment group‐specific changes in tumor growth curves were analyzed using linear regression models. Two‐way ANOVA followed by Tukey post‐tests were used to compare treatment-induced differences in cytokine secretion for each day and assess changes in immune cell infiltration in both the irradiated and non-irradiated tumors across treatment groups. Log-rank (Mantel-Cox) tests were used to detect differences in survival. Statistical significance was defined as *p* < 0.05 and was carried out using GraphPad Prism V.10.

## Results

### Radiotherapy triggers IL-1 signaling

**Fig. 1:**
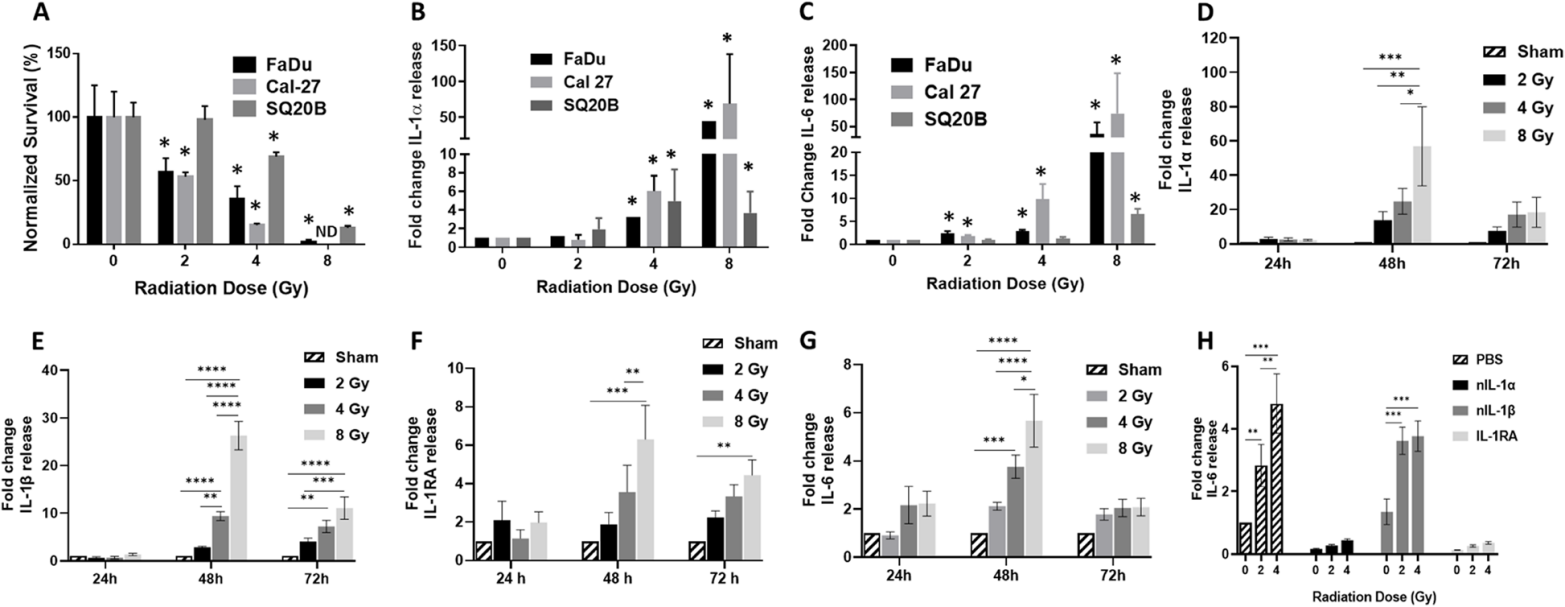
Radiation induces cytokine release. **A**-**C** FaDu, Cal-27 and SQ20B HNSCC cells were exposed to 0 (Sham), 2, 4, and 8 Gy of X-ray radiation and analyzed for clonogenic survival (**A**), IL-1α (**B**) and IL-6 (**C**) 24 h after RT in cell culture media by ELISA. **D**-**G**: Cal-27 HNSCC cells were exposed to 0 (Sham), 2, 4, and 8 Gy of X-ray radiation then cell culture media harvested after the indicated timepoints for analysis of IL-1α (**D**), IL-1β (**E**) IL-1RA (**F**) and IL-6 (**G**) by ELISA. **H**: Cal-27 cells were pretreated with an IL-1 receptor antagonist (IL-1RA) and neutralizing antibodies against IL-1α (nIL-1α) and IL-1β (nIL-1β), exposed to 0 (Sham), 2, 4, and 8 Gy, then IL-6 measured in cell culture media by ELISA. Average values were normalized to Sham control and plotted as fold change. Bars represent the mean of *n* = 3 independent experiments. Error bars represent standard error from the mean. *p < .05; **p < .01; ***p < .001; ****p < .0001. ND: non-detectable

To determine if radiotherapy (RT) releases IL-1 ligands, we first used 3 human HNSCC cell lines (FaDu, Cal-27 and SQ20B) with varying sensitivity to RT (Fig. 1A) and performed clonogenic survival assays 24 h after RT exposure. FaDu and Cal-27 cells were both sensitive to RT at 2, 4 and 8 Gy compared to SHAM-exposed cells (Fig. 1A). SQ20B cells which are known to be radioresistant [25], did not respond to 2 Gy but were sensitive to 4 and 8 Gy (Fig. 1A). IL‐1α was increased (compared to 0 Gy/SHAM) in the cell culture media of all 3 cell lines after 4 and 8 Gy with the highest increase in IL-1α observed at 8 Gy in FaDu and Cal-27 cells (Fig. 1B). IL-6, which is a routinely utilized downstream mechanistic indicator of IL-1R1 signaling [26], was increased (compared to SHAM) in the cell culture media after 2, 4 and 8 Gy for FaDu and Cal-27 and appeared to increase in a dose-dependent manner (Fig. 1C). IL-6 was increased after 8 Gy for SQ20B cells only (Fig. 1C). We then used Cal-27 cells to determine the time course of IL-1α release after RT. Maximum levels of IL-1α were achieved 48 h after RT exposure for all 3 RT doses (Fig. 1D) whereas by 72 h after RT, IL-1α levels either remained the same (e.g. at 2 and 4 Gy) or decreased (at 8 Gy, Fig. 1D). Similar results were observed for IL-1β (Fig. 1E), IL-RA (IL-1 receptor antagonist) which suppresses the IL-1 pathway (Fig. 1F), and IL-6 (Fig. 1G). To inquire if RT triggered IL-1-dependent signaling, we again used IL-6 as an IL-1 signaling endpoint, and pretreated Cal-27 cells with anakinra (recombinant IL-1RA). We observed that anakinra significantly suppressed RT (2 and 4 Gy)-induced IL-6 in Cal-27 cells (Fig. 1H) suggesting that signaling from the IL-1R1 is important for RT-induced IL-6 production. To probe which ligands (IL-1α or IL-1β) may be responsible for activating the IL-1R1, we found that neutralization of IL-1α (but not IL-1β) significantly suppressed RT–induced IL-6 secretion (Fig. 1H), suggesting that IL-1α in particular may be responsible for activating the IL-1R1. Together these results implicate that RT triggers the release of IL-1 ligands and that RT-induced IL-1 signaling is activated by IL-1α release from cancer cells.

### IL-1α activates immune effector cells in vitro

**Fig. 2:**
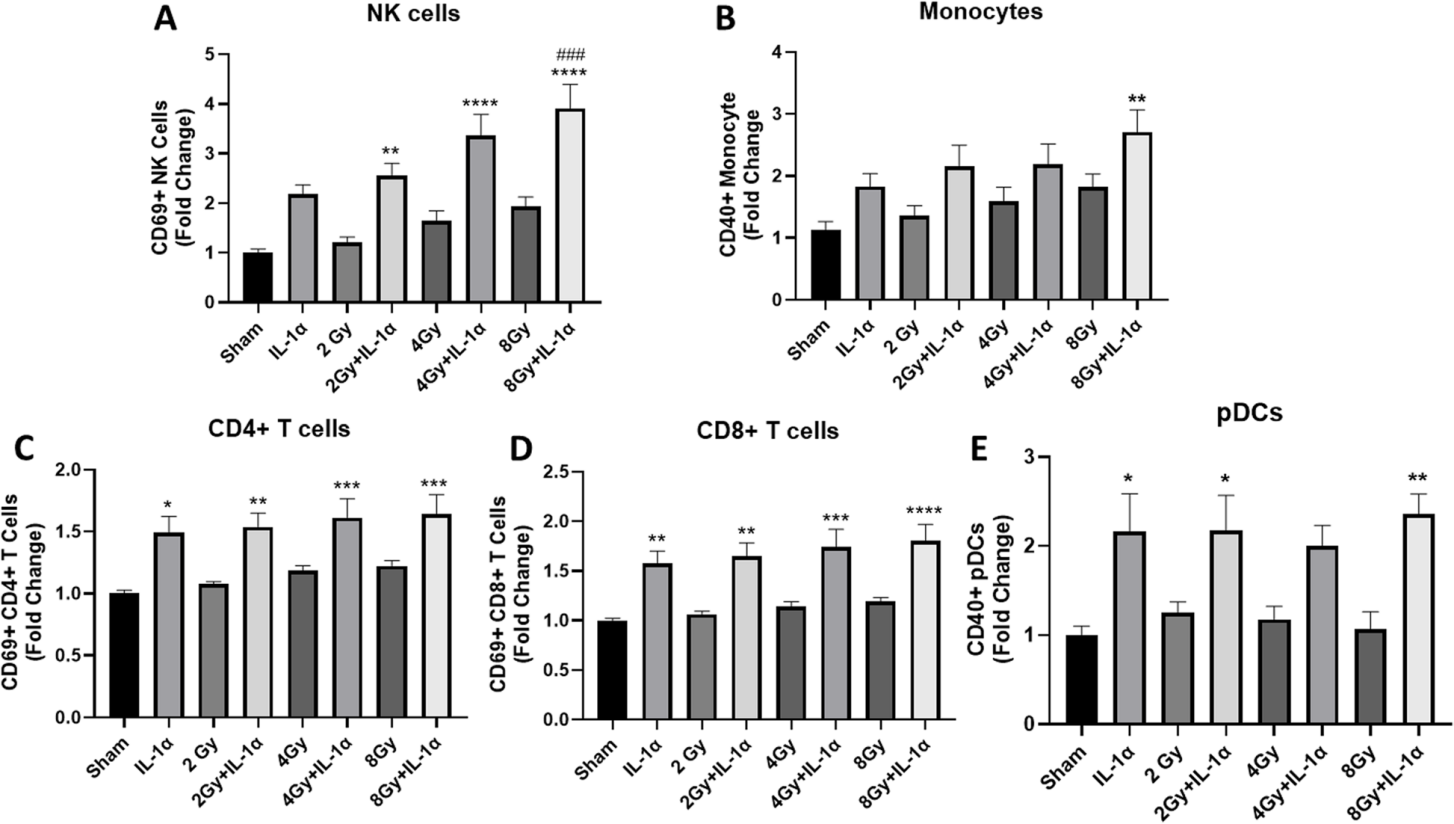
IL-1α activates immune effector cells in vitro. Cal27 HNSCC cells were exposed to 0, 2, 4, and 8 Gy of X-ray radiation, then co-cultured with human peripheral blood mononuclear cells (PBMCs). PBMC/Cal27 co-cultures were treated with recombinant IL-1α for 24 h then analyzed for activated natural killer (NK) cells (**A**), monocytes (**B**), CD4 + T cells (**C**), CD8 + T cells (**D**), and plasmacytoid dendritic cells (pDCs) (**E**) by flow cytometry. Average values were normalized to sham control and plotted as fold change. Bars represent the mean of *n* = 3 independent experiments. Error bars represent SE from the mean. * indicates significant difference from sham control, and # indicate significant difference from rIL-1α. *p < .05; **p < .01; ***p < .001; ****p < .0001

To investigate if exogenous IL-1α would enhance immune cell activation in the presence of RT-exposed cancer cells in vitro, we co-cultured human PBMCs with RT (0–8 Gy)-exposed Cal-27 cells and treated the co-cultures with human recombinant IL-1α (rIL-1α). We found that RT alone (at all doses) did not enhance any of the immune cells tested (Fig. 2A-E). However, we found that all doses of RT in combination rIL-1α significantly increased NK cell activation compared to control/SHAM-treated co-cultures (Fig. 2A). RT at 8 Gy in combination with rIL-1α also significantly increased NK cell activation compared to RT and rIL-1α alone (Fig. 2A). No significant increases in monocyte activation were observed with the exception of 8 Gy + rIL-1α compared to control/SHAM (Fig. 2B). While rIL-1α alone significantly increased the activation of CD4 + T cells (Fig. 2C), CD8 + T cells (Fig. 2D), and pDCs (Fig. 2E) compared to SHAM control cells, we did not observe any enhanced T cell or DC activation in vitro when IL-1α was combined with RT (Fig. 2C-E).

### IL-1αMPs combined with RT induces an anti-tumor response

**Fig. 3:**
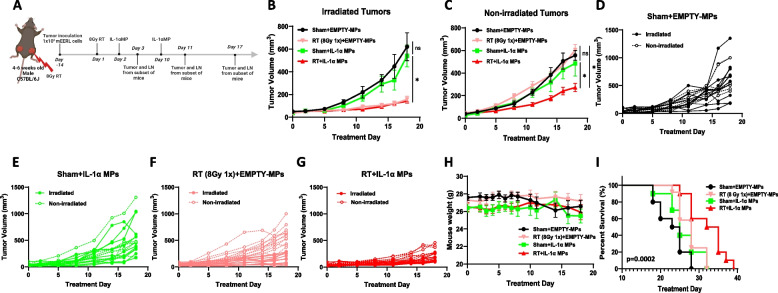
IL-1αMPs combined with radiotherapy (RT) induces an anti-tumor response in non-irradiated tumors. HNSCC (mEERL) bilateral tumor‐bearing C57Bl/6 mice (*n* = 9–10/treatment group) were treated with RT (8 Gy 1X), CPH:SA IL‐1α‐MPs (IL‐1αMPs) or their combination (**A**). Equivalent doses of Blank MPs were administered to Sham mice as a control. RT was administered to the irradiated tumor on Treatment Day 1 followed by two doses of CPH:SA IL‐1α‐MPs (equivalent to 7.5 μg rIL‐1α in 18.75 mg MPs/ dose) intraperitoneally on Treatment days 2 and 10. Tumor growth of both irradiated (**B**) and non-irradiated tumors (**C**) were monitored overtime. **D**-**G**: Tumor growth rate of both irradiated and non-irradiated of individual animals in each of the treatment groups are shown in spaghetti plots (**D**-**G**). Error bars = SEM. *: *p* < 0.05, ns: non-significant

To determine the effects of IL-1α in combination with RT in vivo, we utilized murine recombinant IL-1α encapsulated in CPH:SA (1,6‐bis‐(p‐carboxyphenoxy)‐hexane:sebacic acid (20:80)) microparticles (IL-1αMPs) that we previously reported are non-toxic and releases IL-1α in a slow and sustained manner. Both Empty MPs and IL-1αMPs exhibited an average particle size of approximately 1 µm, with a polydispersity index (PDI) of ~ 0.2. The average zeta potential of the Empty MPs and IL-1αMPs were −16.4 and −17.0 mV respectively. The IL-1αMPs exhibited an average encapsulation efficiency of 33.2% and a drug loading of 0.971 µg per mg of MPs. The MPs’ particle size, zeta potential, and encapsulation efficiency were similar to the IL-1αMPs used in our previous studies [19]. C57Bl/6 mice bearing bilateral mEERL HNSCC tumors were exposed to 8 Gy RT on Treatment Day 1 to one tumor only (proximal); and administered IL-1αMPs (7.5 µg/mouse, i.p) or Blank_CPH:SA (EMPTY MPs) on Treatment Day 2 and Day 10 (Fig. 3A). Administration of IL-1αMPs alone showed no significant changes in tumor growth compared to Sham + EMPTY MPs (control) (Fig. 3B-E). RT exposure significantly suppressed tumor growth in the irradiated (proximal) tumors but not the non-irradiated (distal) tumors compared to control (Fig. 3B,C,D,F). The combination of IL-1MPs and RT significantly slowed down both irradiated (proximal) and non-irradiated (distal) tumor growth (Fig. 3B,C,F,G) and increased survival (based on proximal tumor size endpoint criteria of 15 mm in any dimension) compared to control (Fig. 3I) with no significant changes in weight loss compared to the other treatment groups (Fig. 3H). However, only the combination therapy was able to slowdown non-irradiated (proximal) tumor growth compared to RT alone (Fig. 3C,G). These results suggest that the combination of IL-1αMPs with RT may induce systemic anti-tumor immunity.

### Radiotherapy is associated with lymphodepletion

**Fig. 4:**
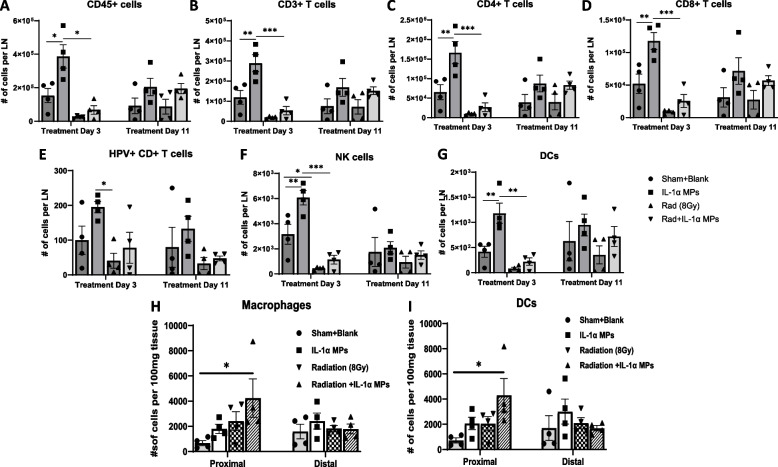
Radiotherapy depletes the immune cell population in the tumor draining lymph nodes. Inguinal lymph nodes near the irradiated tumors were harvested from a subset of mEERL tumor‐bearing C57Bl/6 mice (*n* = 4/treatment group) on Treatment Day 3 (1day after the first dose of IL-1α MPs) and Treatment Day 11 (1day after the second dose of IL-1α MPs). Lymph node homogenates were analyzed by flow cytometry for select immune cell subsets i.e. CD45 + cells (**A**), CD3 + T cells (**B**), CD4 + T cells (**C**), CD8 + T cells (**D**), HPV + CD8 + T cells (**E**), NK cells (**F**), and dendritic cells (DCs) (**G**). Both irradiated and non-irradiated tumors were harvested from a subset of mice from each treatment group. Tumors were homogenized and single cell suspensions were analyzed by flow cytometry for macrophages (**H**) and DCs (**I**). Error bars represent standard error from the mean. *p < .05

On Treatment Day 3 (the following day after the 1st dose of IL-1αMPs, (Fig. 3A), IL-1αMPs significantly increased CD45.2 + cells, CD3 + , CD4 + , and CD8 + T cells, NK cells and DCs in the DLNs compared to control (Fig. 4A-D, F,G). However, immune cells in the DLNs of all mice exposed to RT (regardless of IL-1αMP treatment) were significantly decreased compared to the other treatment groups (Fig. 4A-G). Changes in tumor-infiltrating immune cells were also analyzed from these mice but no significant differences were observed among the treatment groups (data not shown). By Day 11 (the day following the 2nd dose of IL-1αMPs), we observed a general recovery of the immune cell populations in the DLNs from RT-treated mice (Fig. 4A-G) compared to Day 3. The DLN immune cell populations from the RT-treated mice were not different from the control mice; and the DLN immune cell populations from the RT + IL-1αMP-treated mice were not different from the IL-1αMP-treated mice (Fig. 4A-G). The analysis of tumor-infiltrating immune cells at this time point revealed that tumors from the RT + IL-1αMP-treated mice at Day 11 showed significantly higher infiltration of macrophages and DCs (but not other immune cells [data not shown]) in the irradiated (proximal) tumor only compared to the control (Fig. 4H,I). No changes in macrophages, DCs or other immune cells were observed in the unirradiated (distal) tumor (Fig. 4H,I). These results suggest that the effect of IL-1αMPs on immune cells proliferation is severely blunted by RT-indued lymphodepletion but can be restored with time (~ 10 days) to control levels.

### Radiotherapy ± IL-1αMPs induces changes in immune cell recruitment

**Fig. 5:**
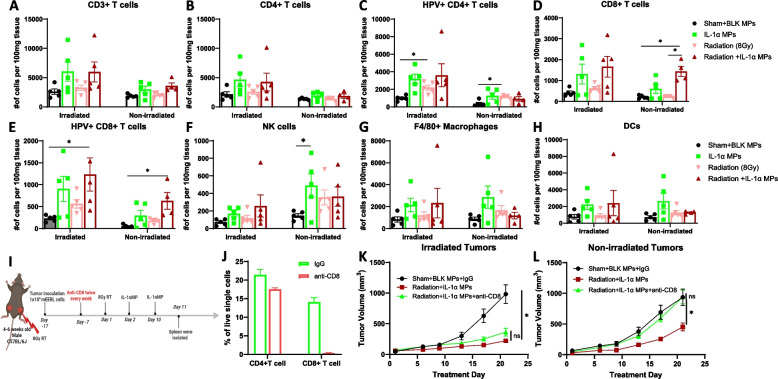
Combination of radiotherapy (RT) and IL-1αMP therapy enhances tumor infiltration of immune effector cells. Irradiated and non-irradiated tumors were harvested from a subset of mice from each treatment group (*n* = 4–5/treatment group) 7 days after the last treatment (Treatment Day 17) from Fig. 3. Tumors were homogenized and single cell suspension were analyzed by flow cytometry for select immune cell subsets such as CD3 + T cells (**A**), CD4 + T cells (**B**), HPV + CD4 + T cells (**C**), CD8 + T cells (**D**), HPV + CD8 + T cells (**E**), NK cells (**F**), macrophages (**G**), and DCs (**H**). HNSCC (mEERL) bilateral tumor‐bearing C57Bl/6 mice (*n* = 10–12/treatment group) were treated with RT (8 Gy 1X) + CPH:SA IL‐1α MPs with or without anti-CD8 antibodies (**I**). Splenic peripheral blood mononuclear cells (PBMCs) were isolated from a subset of mice (*n* = 4) on Treatment Day 11 day and were analyzed by flow cytometry for validation of CD8 + T cell depletion (**J**). Tumor growth of both irradiated (**K**) and non-irradiated tumors (**L**) were monitored overtime. Error bars represent standard error from the mean. *p < .05

To investigate if changes in tumor-infiltrating immune cells were associated with the decreased proximal and distal tumor growth observed in RT + IL-1αMP-treated mice (Fig. 3B,C,G), we waited until significant differences were observed in tumor growth (Fig. 3B,C) and analyzed immune cells from both the irradiated and non-irradiated tumors from a subset of mice (*n* = 4–5/group) on Day 19 (7 days after the last MP treatment). Of all the immune cell populations analyzed (Fig. 5A-H), the irradiated tumors of RT + IL-1αMP-treated mice showed a significant increase in antigen (HPV-E7)-specific CD8 + T cells compared to control (Fig. 5E). Remarkably, we found significantly higher CD8 + T cells (Fig. 5D) cells and HPV-E7 CD8 + T cells (Fig. 5E) cells in the non-irradiated tumors of RT + IL-1αMP-treated mice compared to control suggesting that the anti-tumor effect of RT + IL-1αMP treatment may be associated with increased CD8 + T cells. To confirm this, we investigated the impact of CD8 + T cell depletion on tumor response to RT + IL-1αMP combination therapy. Anti-CD8 antibodies were administered to mice 6 days before the start of RT (Day −7) and continued twice per week for 2 weeks (Fig. 5I). Validation of CD8 + T cell depletion by the depletion antibody from the spleens of treated mice is shown in Fig. 5J. While there was no significant protection in anti-tumor response observed in the irradiated (proximal) tumors (Fig. 5K), depletion of CD8 + T cells completely protected non-irradiated (distal) tumors from the anti-tumor effects of the combination therapy (Fig. 5L). Altogether these data suggest that the combination of IL-1αMPs with RT may induce systemic anti-tumor immunity by increasing the production of CD8 + T cells.

## Discussion

Altogether, our results indicate that the safe addition of IL-1α using CPH:SA-based IL-1α-MP delivery to RT-based protocols may be a promising immunotherapeutic strategy to target local and systemic disease. While the cellular release of activating IL-1 ligands (in addition to other DAMPS) is triggered by RT, IL-1 ligands were of interest here for several reasons. First, it is known that a lack of pre-existing DC activity in tumors impedes the proliferation/activation of TILs (including cytotoxic CD8 + T cells); and on the ability of RT to trigger an anti-tumor immune response and synergize with immunotherapy [18]. IL-1 ligands are able to increase DC maturation and activation [27, 28] which is supported by our in vitro PBMC/cancer cell co-cultures showing IL-1α-induced DC activation (Fig. 2E) and our in vivo results showing DC activation in TDLNs after IL-1αMP treatment (Fig. 4G). We additionally showed that mice exposed to RT + IL-1αMPs generated an increase in CD8 + T cell and antigen-specific (HPV-E7) CD8 + T cell infiltration in non-irradiated (distal) tumors (compared to control, Fig. 5D,E) and the anti-tumor effect of RT + IL-1αMPs on distal tumors was CD8 + T cell dependent (Fig. 5I-L). Second, unlike checkpoint inhibitors that only stimulate T cell activation, IL-1 ligands can activate NK cells (Fig. 2A) [29, 30], in addition to CD4^+^ (Fig. 2C) [31] and CD8^+^ T cells (Fig. 2D) [32–34] suggesting a more broad anti-tumor immune cell profile may be stimulated. Third, clinical studies with rIL-1 ligands (marketed as Dainippon and Immunex) have shown promising results [23]. However, development of dose-related side effects, most notable hypotension, resulted in lessened enthusiasm for this approach which led to our rationale of slow and sustained MP delivery of IL-1α.

Despite RT triggering IL-1α release, RT (alone) did not increase immune cell activation in the PBMC/cancer cell co-cultures in Fig. 2. It is possible that IL-1 ligands were not released in sufficient quantities—which is unlikely since remarkably low levels of IL-1 ligands can trigger immune cell activation [35, 36]. Alternatively, it is likely that RT-induced IL-1 ligands, despite their potent biological activity, are quickly cleared (within 1 h) by the induction of IL-1RA preventing the sustained biologic effect on immune cell activation [35, 36]. Indeed, our results indicate that the release of IL-1RA after RT mimics the same release pattern as IL-1α (Fig. 1D) and IL-1β (Fig. 1E). Immune cells are highly responsive to very small amounts of IL-1 ligands; and IL-1RA levels of over 100-fold molar excess are required to block IL-1 ligand binding to the IL-1R1. In fact, maximal biological responses are observed even when less than 5% of available IL-1R1 are occupied by IL-1 [35, 36]. It is for these reasons that our goal is to safely disrupt the IL-1α/IL-1RA balance by the slow release of IL-1 ligands over time using appropriate delivery vehicles in combination with RT to trigger and sustain maximum systemic anti-tumor immunity.

RT-induced ablation of in-field tumor-infiltrating lymphocytes (TILs) and TILs in the TDLNs is major factor in the disappointing outcomes of RT + immunotherapy studies especially for LA-HNSCC. Despite the favorable results in tumor growth we show with RT + IL-1αMPs (Fig. 3B,C), immune cell depletion in the TDLNs was evident 2 days after RT exposure in our in vivo studies (Fig. 4). However, by 10 days after RT exposure, the immune cells were recovered to respective control levels (Fig. 4) although it is possible that they could have recovered sooner than this time point that we chose to analyze. This brings up the issue of timing or sequencing of RT and immunotherapy. The administration of immunotherapy before, at the same time or immediately after RT may not be wise given that RT-induced lymphodepletion negates the efficacy of most immunotherapies that require lymphocytes for their mechanism of action. Instead finding the optimum immune cell recovery period following RT before administration of immunotherapy would allow for the most efficacious RT/immunotherapy combination response. In support of this we did not observe a notable separation in the RT + IL-1αMP tumor growth curves until Treatment Day 11 (10 days after RT, Fig. 3B,C), which corresponds to when the immune cells had fully recovered to control levels (Fig. 4).

Other factors affecting the efficacy of RT + immunotherapy are RT dose, fractionation protocols and nodal sparing. Monocytes and DCs are more tolerant to low dose (0.5–2 Gy) radiation compared to T cells; however high doses of radiation (above 5 Gy) can lead to substantial cell death [37, 38]. In the current study we used a high RT dose of 8 Gy, which explains why we observed a broad depletion of immune cells (Fig. 4) in the TDLNs in all RT-treated mice 2 days after RT exposure. The other limitation of our approach of using 8 Gy is that this dose is much higher than the standard RT dose for LA-HNSCC which is 2 Gy per day for a total of 70 Gy over 7 weeks. High RT in LA-HNSCC patients is associated with late toxicities related to mucositis, dermatitis, dysphagia. However, our justification for the use of 8 Gy for these studies is the desire to use a single dose of RT to allow a recovery period for circulating immune-effector cells. RT fractionation schedules such as those used in standard protocols for LA-HNSCC (2 Gy/day, 5 fractions/week over 7 weeks) may negatively affect anti-tumor immune response due to the repeated killing of circulating immune effector cells [39]. Perhaps additional studies using single moderate doses of RT in combination with immunotherapy as investigated in the current work would answer important questions about preferred RT dosing and fractionation schedules to combine with immunotherapy.

Lastly, studies have shown that nodal RT can improve control of disease that has spread to the lymph nodes but may kill immune-effector cells residing in those lymph nodes [40, 41]. The incorporation of image-guided targeted RT (e.g. cone beam CT image guidance with treatment planning) to minimize the radiation dose delivered to adjacent non-targeted tissues (including lymph nodes) should partially address this issue. However, previous studies compared tumor growth and T cell infiltration after RT to the tumor versus RT to both tumor and the DLNs. No difference in tumor growth rate was observed, but the proportion and number of CD8 + cells infiltrating the tumor significantly decreased when lymph nodes were included in the radiation field. Moreover, an increase in T-cell chemoattractant and a higher antigen-specific T cell response was noted when DLNs were excluded from the irradiation field and only the tumor was radiated [42]. Clearly, given the opposing roles of lymph nodes in disease dissemination and anti-tumor immune response, more studies are needed to address this issue.

## Conclusions

In summary, our data provide a rationale for the addition of CPH:SA IL-1αMPs to RT which may boost anti-tumor immune response and target both local and systemic disease. This novel immunotherapeutic strategy and may possibly prevent tumor recurrence after surgery from undetected cancer cells in high-risk LA-HNSCC patients.

## Data Availability

Availability of data and materials: The data and materials used and/or analyzed during the current study are available from the corresponding author on reasonable request.

## Abbreviations

IL-1α: Interleukin-1 alpha
IL-1β: Interleukin-1 beta
IL-1RA: Interleukin-1 receptor antagonist
IL-6: Interleukin-6
PBMCs: Peripheral blood mononuclear cells
NK: Natural killer
APC: Antigen-presenting cell
DC: Dendritic cell
pDC: Plasmacytoid dendritic cell
mDC: Monocytic dendritic cells
MYD88: Myeloid differentiation factor 88
ICS: Intracellular cytokine staining
HNSCC: Head and neck squamous cell carcinoma
